# Supplementary material for: TFIP11 promotes replication fork reversal to preserve genome stability
Source: Nat Commun. 2024 Feb 10;15:1262. doi: 10.1038/s41467-024-45684-3 (PMC10858868; doi:10.1038/s41467-024-45684-3)
Supplement: Supplementary file 1 — Supplementary Information [file 41467_2024_45684_MOESM1_ESM.pdf]

Supplementary Information

TFIP11 promotes replication fork reversal to preserve genome stability

Junliang Chen[1,2,3,#], Mingjie Wu[4,#], Yulan Yang[2,#], Chunyan Ruan[2,#], Yi Luo[2], Lizhi Song[2], Ting Wu[2], Jun Huang[1,2], Bing Yang[2], and Ting Liu[1,5,*]

[1]Zhejiang Provincial Key Laboratory of Geriatrics and Geriatrics Institute of Zhejiang Province, Affiliated Zhejiang Hospital, Zhejiang University School of Medicine, 310058, Hangzhou, China

[2]The MOE Key Laboratory of Biosystems Homeostasis & Protection and Zhejiang Provincial Key Laboratory of Cancer Molecular Cell Biology, Life Sciences Institute, Zhejiang University, 310058, Hangzhou, China

[3]Center for Life Sciences, Shaoxing Institute, Zhejiang University, 321000, Shaoxing, China

[4]The Trauma Center, The First Affiliated Hospital, Zhejiang University School of Medicine, 310058, Hangzhou, China

[5]Department of Cell Biology, Zhejiang University School of Medicine, 310058, Hangzhou, China

[#]These authors contribute equally to this work

[*]To whom correspondence should be addressed: liuting518@zju.edu.cn

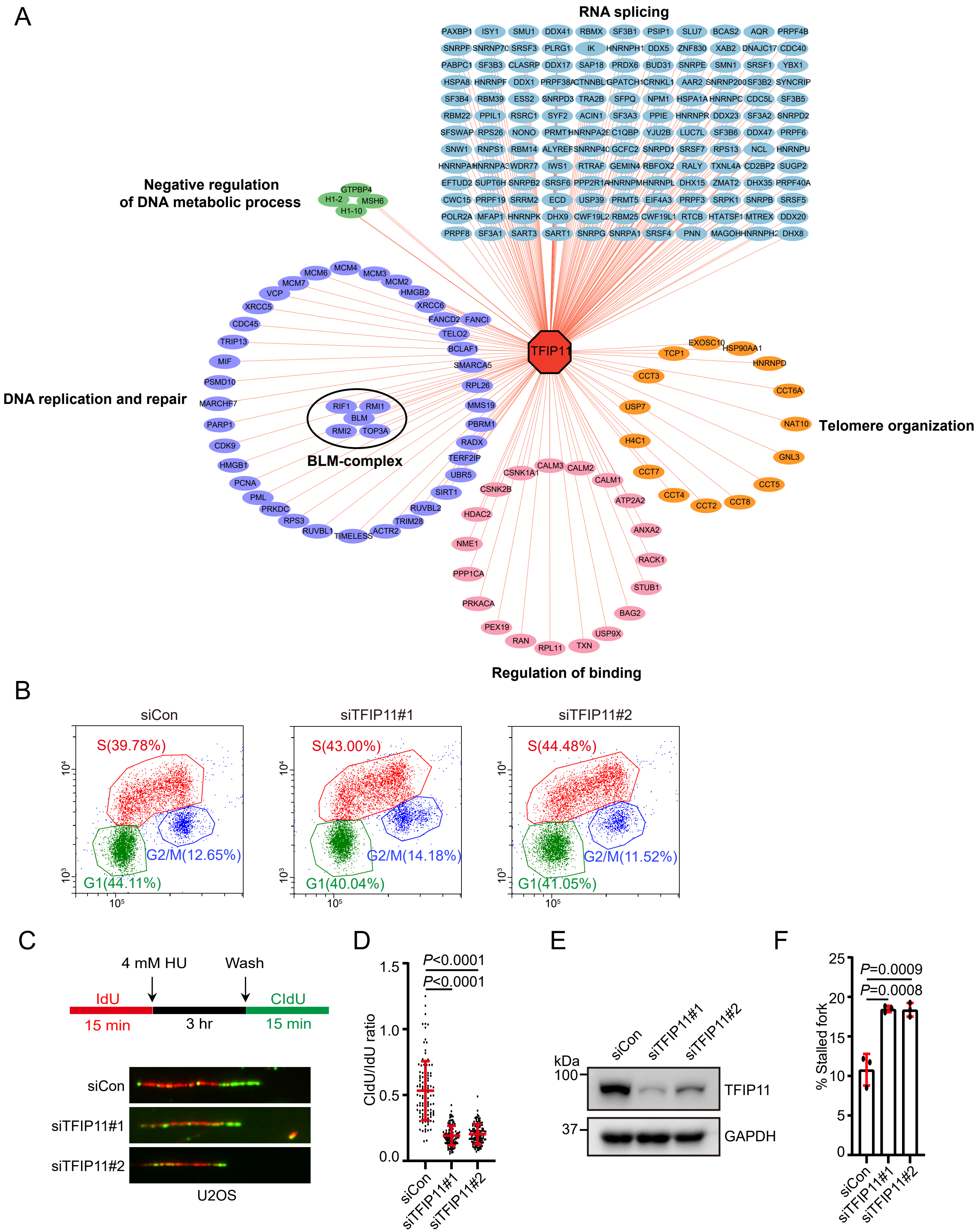
A
RNA splicing
PAXBP1 ISY1 SMU1 DDX41 RBMX SF3B1 PSIP1 SLU7 BCAS2 AQR PRPF4B
SNRPF SNRNP70 SRSF3 PLRG1 IK HNRNPH1 DDX5 ZNF830 XAB2 DNAJC17 CDC40
PABPC1 SF3B3 CLASRP DDX17 SAP18 PRDX6 BUD31 SNRPE SMN1 SRSF1 YBX1
HSPA8 HNRNPF DDX1 PRPF38A CTNNBL1 GPATCH1 CRNKL1 AAR2 SNRNP200 SF3B2 SYNCRIP
SF3B4 RBM39 ESS2 SNRPD3 TRA2B SFPQ NPM1 HSPA1A HNRNPC CDC5L SF3B5
RBM22 PPIL1 RSRC1 SYF2 ACIN1 SF3A3 PPIE HNRNPR DDX23 SF3A2 SNRPD2
SFSWAP RPS26 NONO PRMT1 HNRNPA2B1 C1QBP YJU2B LUC7L SF3B6 DDX47 PRPF6
SNW1 RNPS1 RBM14 ALYREF SNRNP40 GCFC2 SNRPD1 SRSF7 RPS13 NCL HNRNPU
HNRNPA1 HNRNPA3 WDR77 IWS1 RTRAF GEMIN4 RBFOX2 RALY TXNL4A CD2BP2 SUGP2
EFTUD2 SUPT6H SNRPB2 SRSF6 PPP2R1A HNRNPM HNRNPL DHX15 ZMAT2 DHX35 PRPF40A
CWC15 PRPF19 SRRM2 ECD USP39 PRMT5 EIF4A3 PRPF3 SRPK1 SNRPB SRSF5
POLR2A MFAP1 HNRNPK DHX9 CWF19L2 RBM25 CWF19L1 RTCB HTATSF1 MTREX DDX20
PRPF8 SF3A1 SART3 SART1 SNRPG SNRPA1 SRSF4 PNN MAGOH HNRNPH2 DHX8
Negative regulation
of DNA metabolic process
GTPBP4 H1-2 MSH6 H1-10
TFIP11
DNA replication and repair
MCM4 MCM6 MCM3 MCM7 MCM2 VCP HMGB2 XRCC5 XRCC6 FANCD2 FANCI CDC45 TELO2 TRIP13 BCLAF1 MIF SMARCA5 PSMD10 RPL26 MARCHF7 MMS19 PARP1 PBRM1 CDK9 RADX HMGB1 TERF2IP PCNA UBR5 PML SIRT1 PRKDC RUVBL2 RPS3 TRIM28 RUVBL1 TIMELESS ACTR2
RIF1 RMI1 BLM RMI2 TOP3A
BLM-complex
EXOSC10 HSP90AA1 TCP1 HNRNPD CCT3 CCT6A USP7 NAT10 H4C1 GNL3 CCT7 CCT5 CCT4 CCT2 CCT8
Telomere organization
CALM3 CALM2 CSNK1A1 CALM1 CSNK2B ATP2A2 HDAC2 ANXA2 NME1 RACK1 PPP1CA STUB1 PRKACA BAG2 PEX19 USP9X RAN RPL11 TXN
Regulation of binding
B
siCon
S(39.78%)
G2/M(12.65%)
G1(44.11%)
siTFIP11#1
S(43.00%)
G2/M(14.18%)
G1(40.04%)
siTFIP11#2
S(44.48%)
G2/M(11.52%)
G1(41.05%)
10^4
10^3
10^5
C
4 mM HU
Wash
IdU
CldU
15 min
3 hr
15 min
siCon
siTFIP11#1
siTFIP11#2
U2OS
D
P<0.0001
P<0.0001
CldU/IdU ratio
1.5
1.0
0.5
0.0
siCon
siTFIP11#1
siTFIP11#2
E
kDa
siCon
siTFIP11#1
siTFIP11#2
100
TFIP11
37
GAPDH
F
P=0.0009
P=0.0008
% Stalled fork
25
20
15
10
5
0
siCon
siTFIP11#1
siTFIP11#2

Supplementary Figure 1. TFIP11 promotes restart of stalled forks.

(A) Gene ontology (GO) term analysis were performed with Cytoscape software. **(B)** TFIP11 depletion does not affect cell proliferation. U2OS cells were transfected with the indicated siRNAs. 48 h after transfection, BrdU incorporation assays were carried out as described in the Methods section. **(C)** Top: schematic representation of the DNA fiber assay. U2OS cells were labeled with IdU (red) for 15 min, challenged with 4 mM HU for 3 h, and then labeled with CldU (green) for 15 min. Bottom: representative fiber images for each sample are shown. **(D)** Dot plot of CldU/IdU ratios for individual replication forks. Data are representative of three independent experiments. From left, *n* =113, 118, 118 fibers. *P* values were derived from a one-way ANOVA with Tukey's multiple comparisons test. **(E)** Knockdown efficiency of TFIP11 in U2OS cells was confirmed by immunoblotting. **(F)** TFIP11 depletion increases the frequency of fork stalling. HeLa cells were transfected with the indicated siRNAs for 48 hr and analyzed by DNA fiber assay as indicated. The percentage of stalled forks (IdU^{+} $CldU^{-}$) was determined. Data are representative of three independent experiments. From left, *n* = 992, 1350, 1134 fibers. *P* values were derived from a one-way ANOVA with Tukey's multiple comparisons test. Source data are provided as a Source Data file.

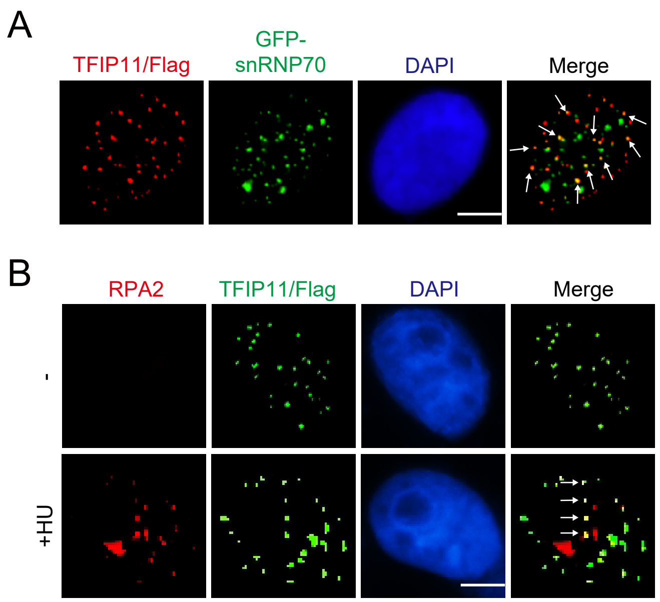
A
TFIP11/Flag
GFP-
snRNP70
DAPI
Merge
B
RPA2
TFIP11/Flag
DAPI
Merge
-
+HU

Supplementary Figure 2. TFIP11 accumulates at stalled forks.

(A) TFIP11 partially co-localizes with the splicing factor snRNP70. SF-TFIP11 knock-in HeLa cells were transfected with the indicated plasmids. 24 hr after transfection, cells were subjected to immunostaining. Arrow indicates colocalization of TFIP11 and SNRNP70. Scale bar, 10 μm. **(B)** A small fraction of TFIP11 is recruited to stalled forks where it colocalizes with RPA. SF-TFIP11 knock-in HeLa cells were transfected with the indicated plasmids. 24 hr after transfection, cells were treated with 4 mM HU for 3 h and then subjected to immunostaining. Arrow indicates colocalization of TFIP11 and RPA2. Scale bar, 10 μm. Source data are provided as a Source Data file.

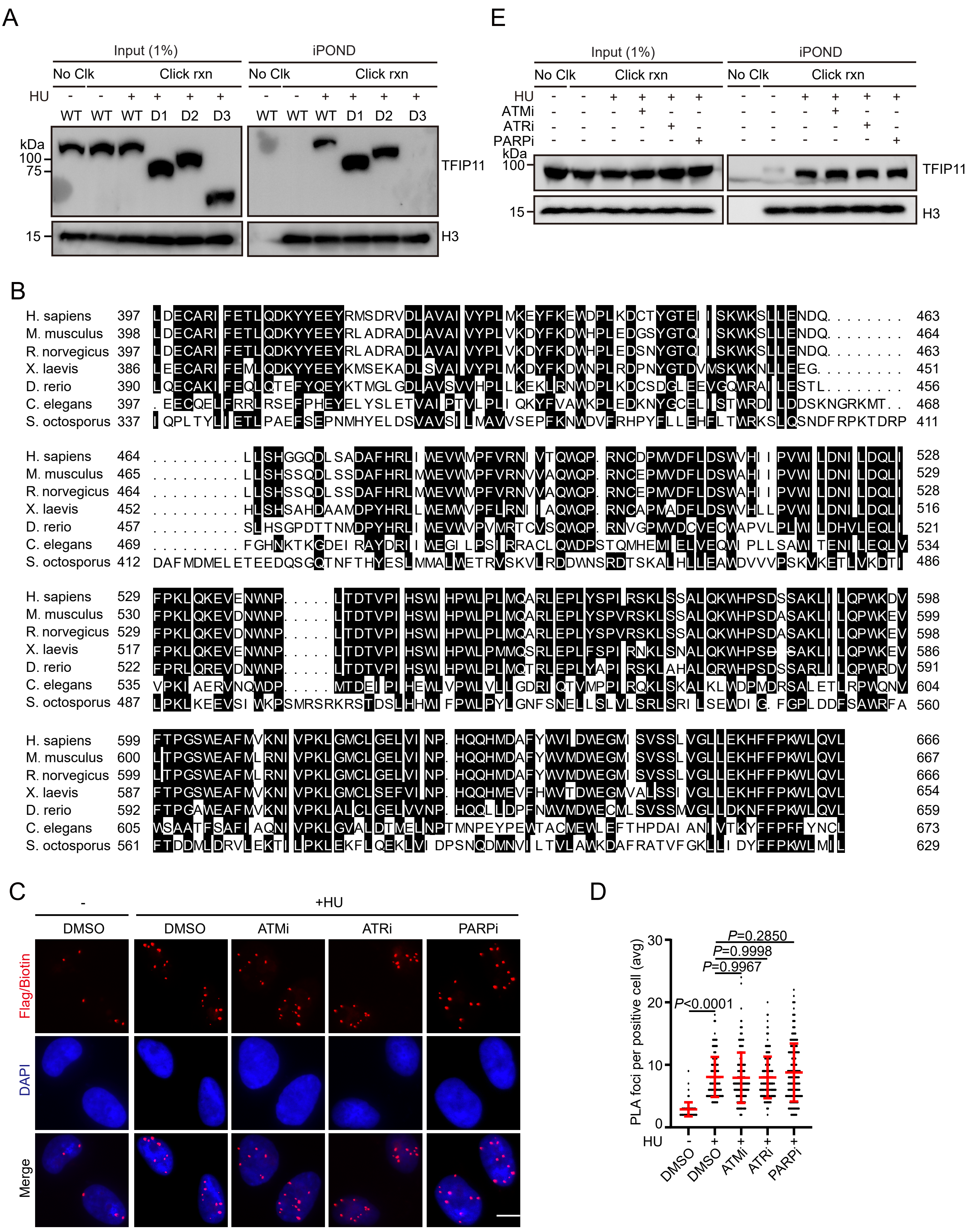
A
Input (1%)
iPOND
No Clk
Click rxn
HU
WT
D1
D2
D3
kDa
100
75
15
TFIP11
H3
E
ATMi
ATRi
PARPi
B
H. sapiens 397 LDECARIFETLQDKYYEEYRMSDRVDLAVAIVYPLMKEYFKEWDPLKDCTYGTEIISKWKSLLENDQ......... 463
M. musculus 398 LDECARIFETLQDKYYEEYRLADRADLAVAIVYPLVKDYFKDWHPLEDGSYGTQIISKWKSLLENDQ......... 464
R. norvegicus 397 LDECARIFETLQDKYYEEYRLADRADLAVAIVYPLVKDYFKDWHPLEDSNYGTQIISKWKSLLENDQ......... 463
X. laevis 386 LEECARIFEMLQDKYYEEYKMSEKADLSVAIVYPLMKDYFKDWNPLRDPNYGTDVMSKWKNLLEEG.......... 451
D. rerio 390 LQECAKIFEQLQTEFYQEYKTMGLGDLAVSVVHPLLKEKLRNWDPLKDCSDGLEEVGQWRAILESTL......... 456
C. elegans 397 .EECQELFRRLRSEFPHEYELYSLETVAIPTVLPLIQKYFVAWKPLEDKNYGCELISTWRDILDDSKNGRKMT.. 468
S. octosporus 337 IQPLTYLIETLPAEFSEPNMHYELDSVAVSILMAVVSEPFKNWDVFRHPYFLLEHFLTWRKSLQSNDFRPKTDRP 411
H. sapiens 464 ..........LLSHGGQDLSADAFHRLIWEVWMPFVRNIVTQWQPRNCDPMVDFLDSWVHIIPVWILDNILDQLI 528
M. musculus 465 ..........LLSHSSQDLSSDAFHRLMWEVWMPFVRNVVAQWQPRNCEPMVDFLDSWAHIIPVWILDNILDQLI 529
R. norvegicus 464 ..........LLSHSSQDLSSDAFHRLMWEVWMPFVRNVVAQWQPRNCEPMVDFLDSWAHIIPVWILDNILDQLI 528
X. laevis 452 ..........HLSHSAHDAAMDPYHRLLWEMWVPFLRNIIAQWQPRNCAPMADFLDSWVHLLPVWILDNILDQLI 516
D. rerio 457 ..........SLHSGPDTTNMDPYHRLIWEVWVPVMRTCVSQWQPRNVGPMVDCVECWAPVLPLWILDHVLEQLI 521
C. elegans 469 .........FGHNKTKGDEIRAYDRIIWEGILPSIRRACLQWDPSTQMHEMIELVEQWIPLLSAWITENILEQLV 534
S. octosporus 412 DAFMDMELETEEDQSGQTNFTHYESLMMALWETRVSKVLRDDWNSRDTSKALHLLEAWDVVVPSKVKETLVKDTI 486
H. sapiens 529 FPKLQKEVENWNP.....LTDTVPIHSWIHPWLPLMQARLEPLYSPIRSKLSSALQKWHPSDSSAKLILQPWKDV 598
M. musculus 530 FPKLQKEVDNWNP.....LTDTVPIHSWIHPWLPLMQARLEPLYSPVRSKLSSALQKWHPSDASAKLILQPWKEV 599
R. norvegicus 529 FPKLQKEVDNWNP.....LTDTVPIHSWIHPWLPLMQARLEPLYSPVRSKLSSALQKWHPSDASAKLILQPWKEV 598
X. laevis 517 FPKLQKEVENWNP.....LTDTVPIHSWIHPWLPMMQSRLEPLFSPIRNKLSNALQKWHPSD SAKLILQPWKEV 586
D. rerio 522 FPRLQREVDNWNP.....LTDTVPIHSWIHPWLPLMQTRLEPLYAPIRSKLAHALQRWHPSDSSARLILQPWRDV 591
C. elegans 535 VPKIAERVNQWDP.....MTDEIPIHEWLVPWLVLLGDRIQTVMPPIRQKLSKALKLWDPMDRSALETLRPWQNV 604
S. octosporus 487 LPKLKEEVSIWKPSMRSRKRSTDSLHHWIFPWLPYLGNFSNELLSLVLSRLSRILSEWDIG.FGPLDDFSAWRFA 560
H. sapiens 599 FTPGSWEAFMVKNIVPKLGMCLGELVINP.HQQHMDAFYWVIDWEGMISVSSLVGLLEKHFFPKWLQVL 666
M. musculus 600 LTPGSWEAFMLRNIVPKLGMCLGELVINP.HQQHMDAFYWVMDWEGMISVSSLVGLLEKHFFPKWLQVL 667
R. norvegicus 599 LTPGSWEAFMLRNIVPKLGMCLGELVINP.HQQHMDAFYWVMDWEGMISVSSLVGLLEKHFFPKWLQVL 666
X. laevis 587 FTPGSWEAFMVKNIVPKLGMCLSEFVINP.HQQHMEVFHWVTDWEGMVALSSIVGLLEKHFFPKWLQVL 654
D. rerio 592 FTPGAWEAFMVKNIVPKLALCLGELVVNP.HQQLLDPFNWVMDWECMLSVSSMVGLLDKNFFPKWLQVL 659
C. elegans 605 WSAATFSAFIACNIVPKLGVALDTMELNPTMNPEYPEWTACMEWLEFTHPDAIANIVTKYFFPFFYNCL 673
S. octosporus 561 FTDDMLDRVLEKTILPKLEKFLQEKLVIDPSNQDMNVILTVLAWKDAFRATVFGKLLIDYFFPKWLMIL 629
C
-
+HU
DMSO
ATMi
ATRi
PARPi
Flag/Biotin
DAPI
Merge
D
PLA foci per positive cell (avg)
30
20
10
0
P=0.2850
P=0.9998
P=0.9967
P<0.0001
HU
DMSO
ATMi
ATRi
PARPi

Supplementary Figure 3. TFIP11 recruitment to stalled forks is independent on ATM/ATR/PARP1.

(A) Input and iPOND samples were analyzed by Western blotting. HEK293T cells were labeled with 10 μM EdU for 15 min. The cells were then challenged with 4 mM HU for 3 h prior to crosslinking with 1% formaldehyde. No Clk, no-click samples; rxn, reaction. **(B)** Alignment of the GCFC domain of TFIP11 proteins from different species. **(C-D)** SF-TFIP11 knock-in HeLa cells were labeled with 10 μM EdU for 15 min, left untreated or treated with 4 mM HU for 3 h in the presence of ATM inhibitor (10 μM KU-60019), ATR inhibitor (5 μM VE-821) or PARP1 inhibitor (10 μM Olaparib), and subjected to PLA with anti-Flag and anti-biotin antibodies. Representative images of PLA foci (red) were shown in (C). DNA was stained with DAPI. Scale bar, 10 μm. Quantification of the average number of PLA foci per focus positive cell (D). Data represent means ± SD from three independent experiments. From left, $n = 168, 189, 168, 168, 168$ cells. *P* values were derived from a one-way ANOVA with Tukey's multiple comparisons test. **(E)** Input and iPOND samples were analyzed by Western blotting. HeLa cells were labeled with 10 μM EdU for 15 min, left untreated or treated with 4 mM HU for 3 h in the presence of ATM inhibitor (10 μM KU-60019), ATR inhibitor (5 μM VE-821) or PARP1 inhibitor (10 μM Olaparib) prior to crosslinking with 1% formaldehyde. Source data are provided as a Source Data file.

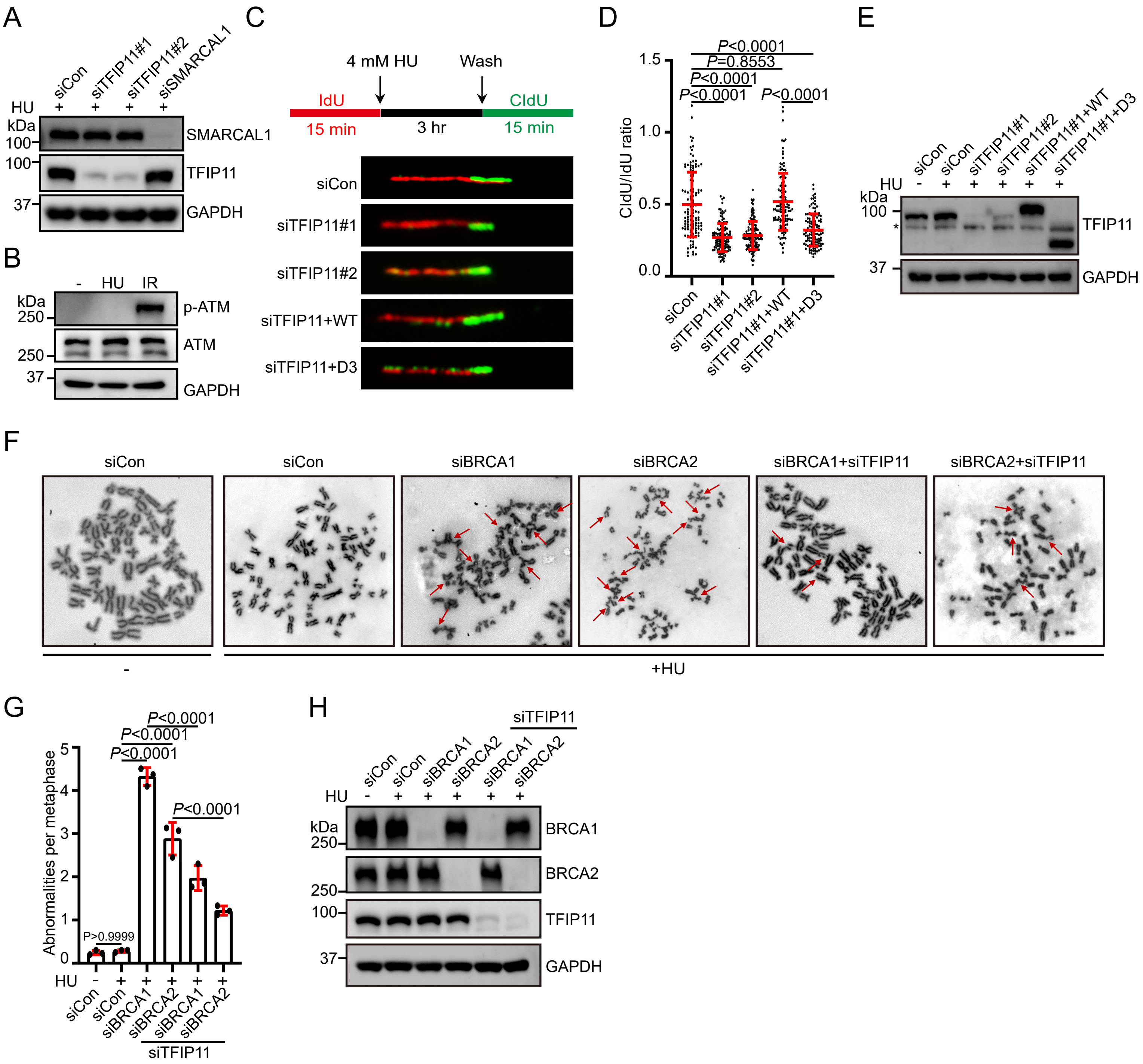
A
siCon
siTFIP11#1
siTFIP11#2
siSMARCAL1
HU
kDa
100
SMARCAL1
TFIP11
37
GAPDH
B
-
HU
IR
250
p-ATM
ATM
GAPDH
C
4 mM HU
Wash
IdU
15 min
3 hr
CldU
siTFIP11+WT
siTFIP11+D3
D
P<0.0001
P=0.8553
CldU/IdU ratio
siTFIP11#1+WT
siTFIP11#1+D3
E
*
F
siBRCA1
siBRCA2
siBRCA1+siTFIP11
siBRCA2+siTFIP11
+HU
G
Abnormalities per metaphase
P>0.9999
siTFIP11
H
BRCA1
BRCA2

Supplementary Figure 4. TFIP11 promotes replication fork reversal and restart.

(**A**) Knockdown efficiency of TFIP11 and SMARCAL1. (**B**) X-ray but not HU treatment induces DSB formation. HeLa cells were either treated with 10 Gy X-ray and allowed 3 h to recover or treated with 4 mM HU for 3 h. Cells were then harvested and subjected to western blotting. **(C)** Top: schematic representation of the DNA fiber assay. HeLa cells were labeled with IdU (red) for 15 min, challenged with 4 mM HU for 3 h, and then labeled with CldU (green) for 15 min. Bottom: representative fiber images for each sample are shown. **(D)** Dot plot of CldU/IdU ratios for individual replication forks. Data represent means ± SD from three independent experiments. From left, $n = 121, 120, 134, 120, 119$ fibers. *P* values were derived from a one-way ANOVA with Tukey's multiple comparisons test. **(E)** Western blot analysis of TFIP11 expression. Asterisk indicates a non-specific band. **(F-G)** Analysis of chromosomal aberrations in HeLa cells transfected with indicated siRNAs upon a 4 mM HU treatment for 5 h. Representative images of metaphase spreads from control or HU-treated HeLa cells transfected with indicated siRNAs (F). Chromosomal aberrations are indicated by arrows. Data are represented as the mean ± SD from three independent experiments (G). From left, $n = 100, 100, 100, 100, 100, 99$ metaphases. *P* values were derived from a one-way ANOVA with Tukey's multiple comparisons test. **(H)** Knockdown efficiency was examined by immunoblotting. Source data are provided as a Source Data file.

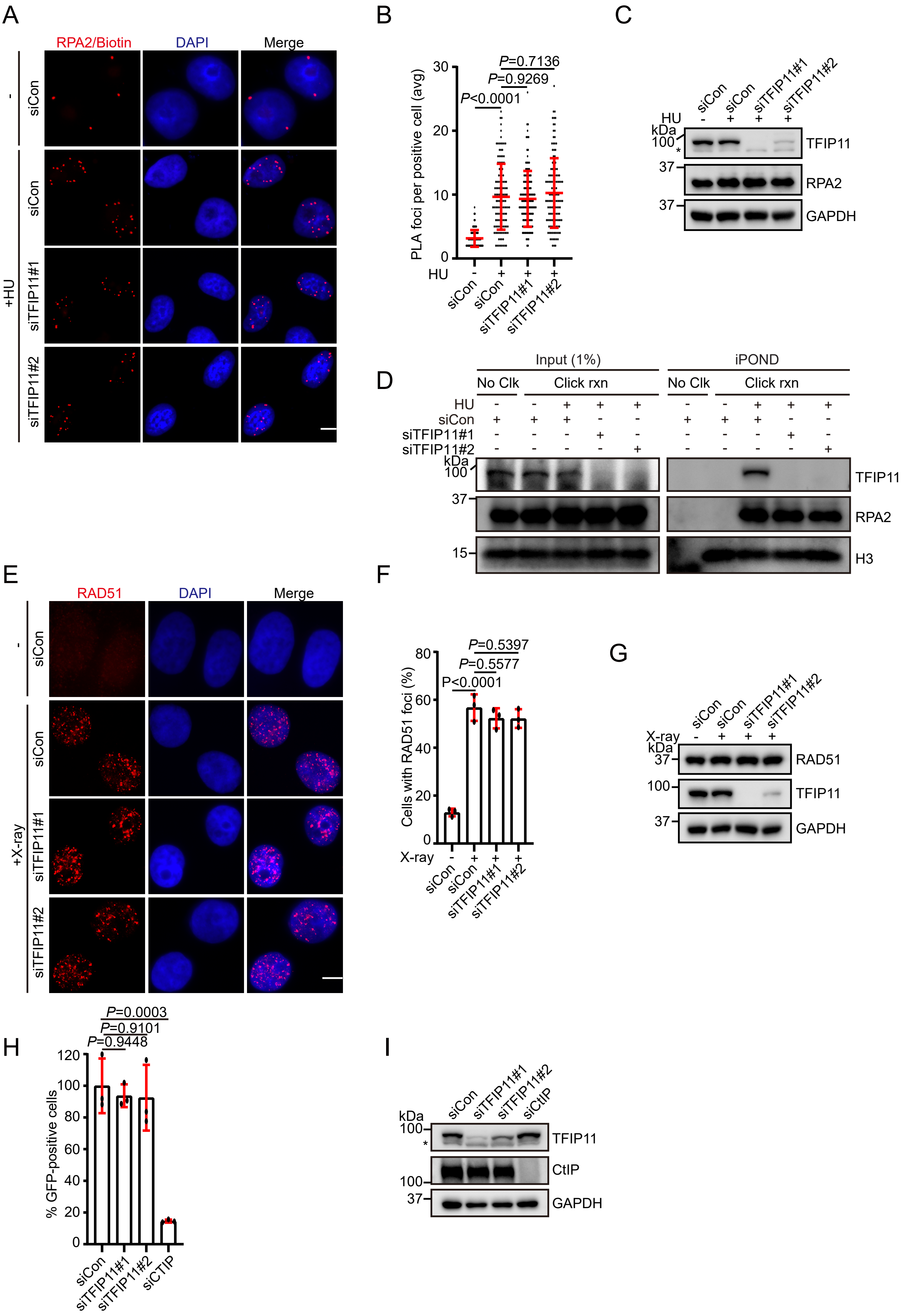
A
RPA2/Biotin
DAPI
Merge
-
siCon
+HU
siCon
siTFIP11#1
siTFIP11#2
B
PLA foci per positive cell (avg)
30
20
10
0
P=0.7136
P=0.9269
P<0.0001
HU
-
+
+
+
siCon
siCon
siTFIP11#1
siTFIP11#2
C
HU
-
+
+
+
siCon
siCon
siTFIP11#1
siTFIP11#2
kDa
100
*
37
37
TFIP11
RPA2
GAPDH
D
Input (1%)
iPOND
No Clk
Click rxn
No Clk
Click rxn
HU
siCon
siTFIP11#1
siTFIP11#2
kDa
100
37
15
TFIP11
RPA2
H3
E
RAD51
DAPI
Merge
-
siCon
+X-ray
siCon
siTFIP11#1
siTFIP11#2
F
Cells with RAD51 foci (%)
80
60
40
20
0
P=0.5397
P=0.5577
P<0.0001
X-ray
-
+
+
+
siCon
siCon
siTFIP11#1
siTFIP11#2
G
X-ray
-
+
+
+
siCon
siCon
siTFIP11#1
siTFIP11#2
kDa
37
100
37
RAD51
TFIP11
GAPDH
H
% GFP-positive cells
120
100
80
60
40
20
0
P=0.0003
P=0.9101
P=0.9448
siCon
siTFIP11#1
siTFIP11#2
siCTIP
I
siCon
siTFIP11#1
siTFIP11#2
siCtIP
kDa
100
*
100
37
TFIP11
CtIP
GAPDH

Supplementary Figure 5. TFIP11 is not required for RAD51-mediated HR repair.

(A-C) TFIP11 is not required for RPA recruitment to stalled forks. HeLa cells were labeled with 10 μM EdU for 15 min before treatment with 2 mM HU for 2 h. PLA was conducted with anti-RPA2 and anti-biotin antibodies. Representative images of PLA foci (red) are shown in (A). DNA was stained with DAPI. Scale bar, 10 μm. Quantification of the average number of PLA foci per focus-positive cell (B). Data represent means ± SD from three independent experiments. From left, $n = 126$, 126, 126, 126 cells. *P* values were derived from a one-way ANOVA with Tukey's multiple comparisons test. Knockdown efficiency was confirmed by immunoblotting (C). Asterisk indicates a non-specific band. **(D)** Input and iPOND samples were analyzed by Western blotting. **(E-G)** TFIP11 is not required for X-ray-induced RAD51 foci formation. HeLa cells were mock treated or treated with X-ray (10 Gy) and were allowed to recover for 6 h before being fixed and processed for RAD51 immunofluorescence. Representative RAD51 foci were shown in (E). Results are the average of three independent experiments and are presented as means ± SD (F). From left, $n = 628$, 560, 520, 680 cells. *P* values were derived from a one-way ANOVA with Tukey's multiple comparisons test. Knockdown efficiency was confirmed by immunoblotting (G). **(H-I)** TFIP11 depletion does not affect HR repair. U2OS DR-GFP cells transfected with indicated siRNAs were electroporated with an I-SceI expression plasmid. 48 h after electroporation, cells were harvested and assayed for GFP expression by flow cytometry analysis. Results are presented as means ± SD from three independent experiments. ns, not significant; $^{**}P<0.\ 01$, one-way ANOVA. Knockdown efficiency was examined by immunoblotting (I). Asterisk indicates a non-specific band. Source data are provided as a Source Data file.

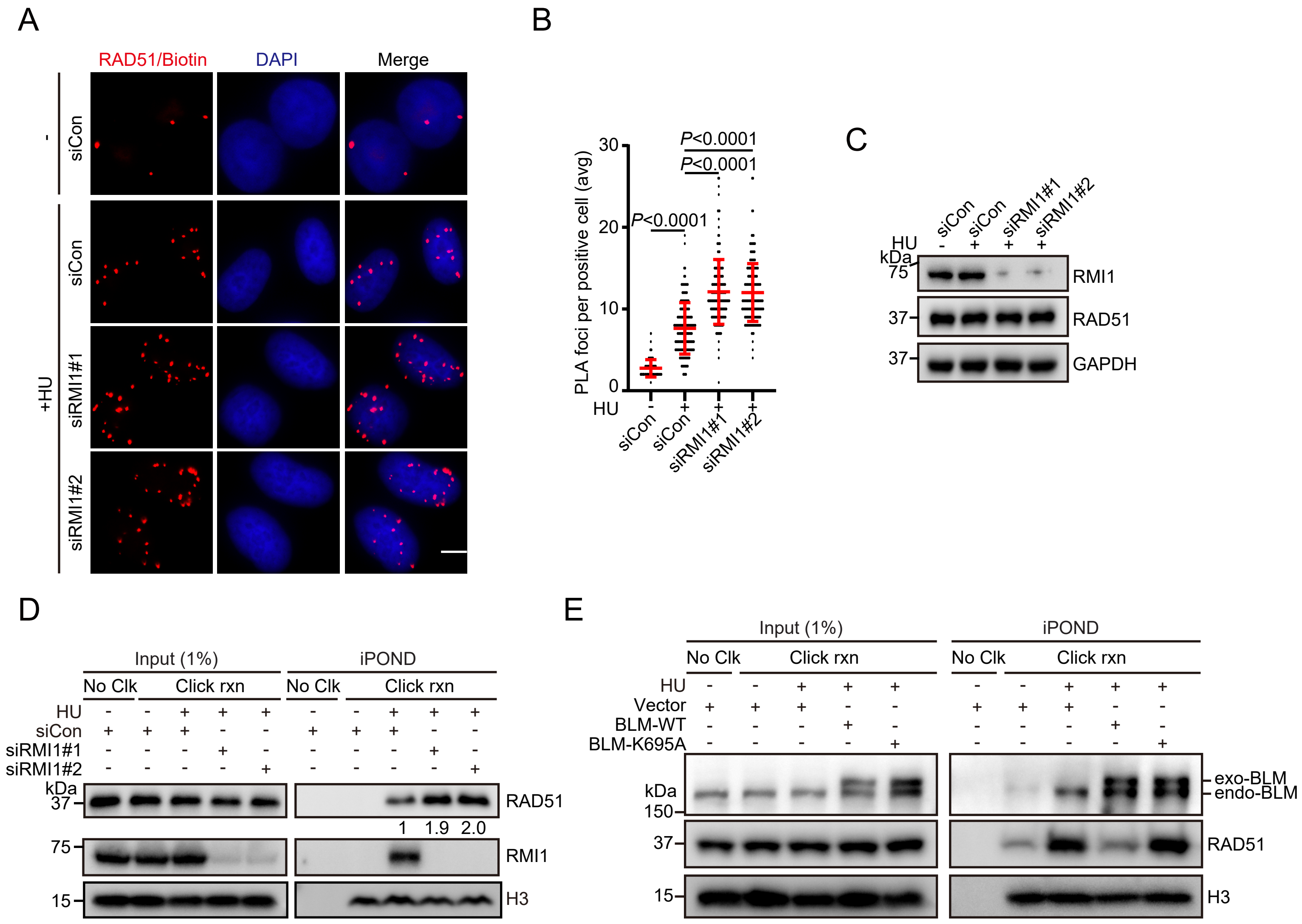
A
RAD51/Biotin
DAPI
Merge
-
siCon
+HU
siCon
siRMI1#1
siRMI1#2
B
PLA foci per positive cell (avg)
P<0.0001
P<0.0001
P<0.0001
HU
siCon
siCon
siRMI1#1
siRMI1#2
C
HU
kDa
siCon
siCon
siRMI1#1
siRMI1#2
RMI1
RAD51
GAPDH
D
Input (1%)
iPOND
No Clk
Click rxn
HU
siCon
siRMI1#1
siRMI1#2
kDa
RAD51
1 1.9 2.0
RMI1
H3
E
Input (1%)
iPOND
No Clk
Click rxn
HU
Vector
BLM-WT
BLM-K695A
kDa
exo-BLM
endo-BLM
RAD51
H3

Supplementary Figure 6. BLM limits excessive accumulation of RAD51 at stalled forks.

(A-C) HeLa cells were labeled with 10 μM EdU for 15 min before treatment with 4 mM HU for 3 h. PLA was conducted with anti-RAD51 and anti-biotin antibodies. Representative images of PLA foci (red) were shown in (A). DNA was stained with DAPI. Scale bar, 10 μm. Quantification of the average number of PLA foci per focus positive cell (B). Data represent means ± SD from three independent experiments. From left, $n = 168$, 168, 168, 170 cells. P values were derived from a one-way ANOVA with Tukey's multiple comparisons test. Knockdown efficiency was confirmed by immunoblotting (C). **(D)** Input and iPOND samples were analyzed by Western blotting. **(E)** Input and iPOND samples were analyzed by Western blotting. Source data are provided as a Source Data file.

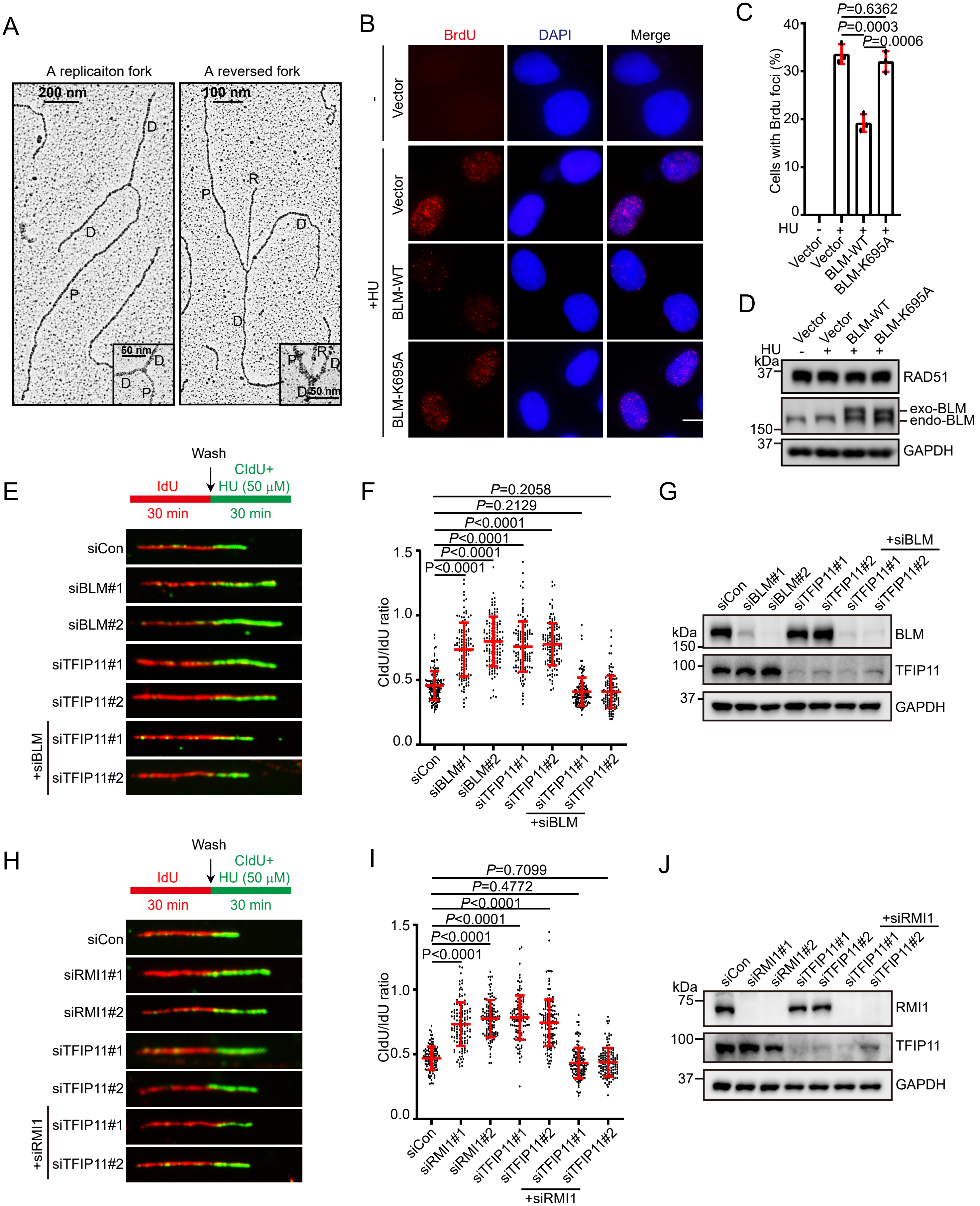
A
A replicaiton fork
A reversed fork
200 nm
100 nm
50 nm
D
P
R
B
BrdU
DAPI
Merge
-
+HU
Vector
BLM-WT
BLM-K695A
C
P=0.6362
P=0.0003
P=0.0006
Cells with Brdu foci (%)
40
30
20
10
0
HU
Vector
Vector
BLM-WT
BLM-K695A
D
HU
kDa
37
RAD51
exo-BLM
endo-BLM
150
GAPDH
E
Wash
IdU
CldU+
HU (50 μM)
30 min
30 min
siCon
siBLM#1
siBLM#2
siTFIP11#1
siTFIP11#2
+siBLM
F
P=0.2058
P=0.2129
P<0.0001
P<0.0001
P<0.0001
P<0.0001
CldU/IdU ratio
1.5
1.0
0.5
0.0
siCon
siBLM#1
siBLM#2
siTFIP11#1
siTFIP11#2
siTFIP11#1
siTFIP11#2
+siBLM
G
+siBLM
siCon
siBLM#1
siBLM#2
siTFIP11#1
siTFIP11#2
siTFIP11#1
siTFIP11#2
kDa
150
BLM
100
TFIP11
37
GAPDH
H
Wash
IdU
CldU+
HU (50 μM)
30 min
30 min
siCon
siRMI1#1
siRMI1#2
siTFIP11#1
siTFIP11#2
+siRMI1
I
P=0.7099
P=0.4772
P<0.0001
P<0.0001
P<0.0001
P<0.0001
CldU/IdU ratio
1.5
1.0
0.5
0.0
siCon
siRMI1#1
siRMI1#2
siTFIP11#1
siTFIP11#2
siTFIP11#1
siTFIP11#2
+siRMI1
J
+siRMI1
siCon
siRMI1#1
siRMI1#2
siTFIP11#1
siTFIP11#2
siTFIP11#1
siTFIP11#2
kDa
75
RMI1
100
TFIP11
37
GAPDH

Supplementary Figure 7. TFIP11 antagonizes the BLM complex to promotes fork slowing.

(A) Electron micrograph of a representative replication fork or reversed replication fork. P indicates the parental duplex, D indicates daughter duplexes, and R indicates the regressed arm. **(B-D)** Overexpression of BLM results in a decrease in ssDNA exposure of nascent DNA strands after HU treatment. HeLa cells were labeled with 10 μM BrdU for 15 min before treatment with 4 mM HU for 3 h. Cells were then fixed and stained with antibody against BrdU under native conditions. Representative BrdU foci were shown in (B). DNA was stained with DAPI. Scale bar, 10 μm. Quantification of BrdU foci (C). Cells with more than 5 BrdU foci were considered positive. Data represent mean ± SD from three independent experiments. From left, $n = 700, 711, 742, 710$ cells. P values were derived from a one-way ANOVA with Tukey's multiple comparisons test. Western blot analysis of BLM expression in the indicated cells (D). **(E, H)** Top: schematic representation of the DNA fiber assay. HeLa cells were labeled with IdU for 30 min, and subsequently with CldU in the presence of HU for 30 min. Bottom: representative fiber images for each sample are shown. **(F)** Dot plot of CldU/IdU ratios for individual replication forks. Data represent mean ± SD from three independent experiments. From left, $n = 134, 113, 122, 126, 120, 128, 123$ fibers. P values were derived from a one-way ANOVA with Tukey's multiple comparisons test. **(I)** Dot plot of CldU/IdU ratios for individual replication forks. Data represent mean ± SD from three independent experiments. From left, $n = 110, 121, 120, 115, 138, 121, 124$ fibers. P values were derived from a one-way ANOVA with Tukey's multiple comparisons test. **(G, J)** Knockdown efficiency was examined by immunoblotting. Source data are provided as a Source Data file.

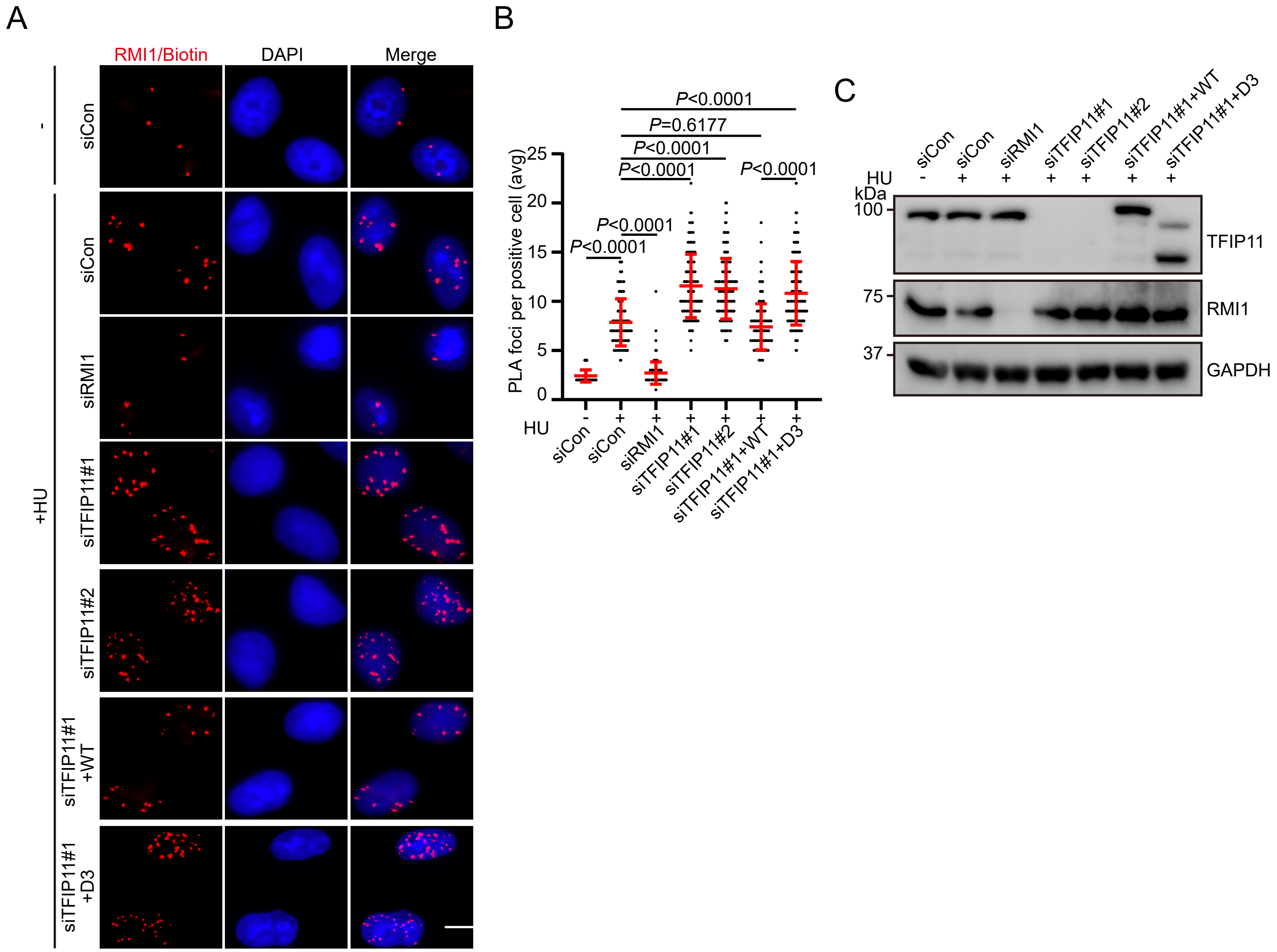

A
RMI1/Biotin
DAPI
Merge
-
siCon
+HU
siCon
siRMI1
siTFIP11#1
siTFIP11#2
siTFIP11#1 +WT
siTFIP11#1 +D3
B
P<0.0001
P=0.6177
P<0.0001
P<0.0001
P<0.0001
P<0.0001
P<0.0001
PLA foci per positive cell (avg)
25
20
15
10
5
0
HU
- + + + + + +
siCon
siCon
siRMI1
siTFIP11#1
siTFIP11#2
siTFIP11#1+WT
siTFIP11#1+D3
C
HU
- + + + + + +
siCon
siCon
siRMI1
siTFIP11#1
siTFIP11#2
siTFIP11#1+WT
siTFIP11#1+D3
kDa
100
75
37
TFIP11
RMI1
GAPDH

Supplementary Figure 8. TFIP11 modulates RMI1 association with stalled forks.

(A-C) TFIP11 depletion increases the numbers of RMI1/biotin PLA foci. HeLa cells were labeled with 10 μM EdU for 15 min before treatment with 4 mM HU for 3 h. PLA was conducted with anti-RMI1 and anti-biotin antibodies. Representative images of PLA foci (red) were shown in (A). DNA was stained with DAPI. Scale bar, 10 μm. Quantification of the average number of PLA foci per focus-positive cell (B). Data represent mean ± SD from three independent experiments. From left, $n = 168, 168, 182, 168, 168, 168, 168$ cells. P values were derived from a one-way ANOVA with Tukey's multiple comparisons test. Knockdown efficiency was confirmed by immunoblotting (C). Asterisk indicates a non-specific band. Source data are provided as a Source Data file.

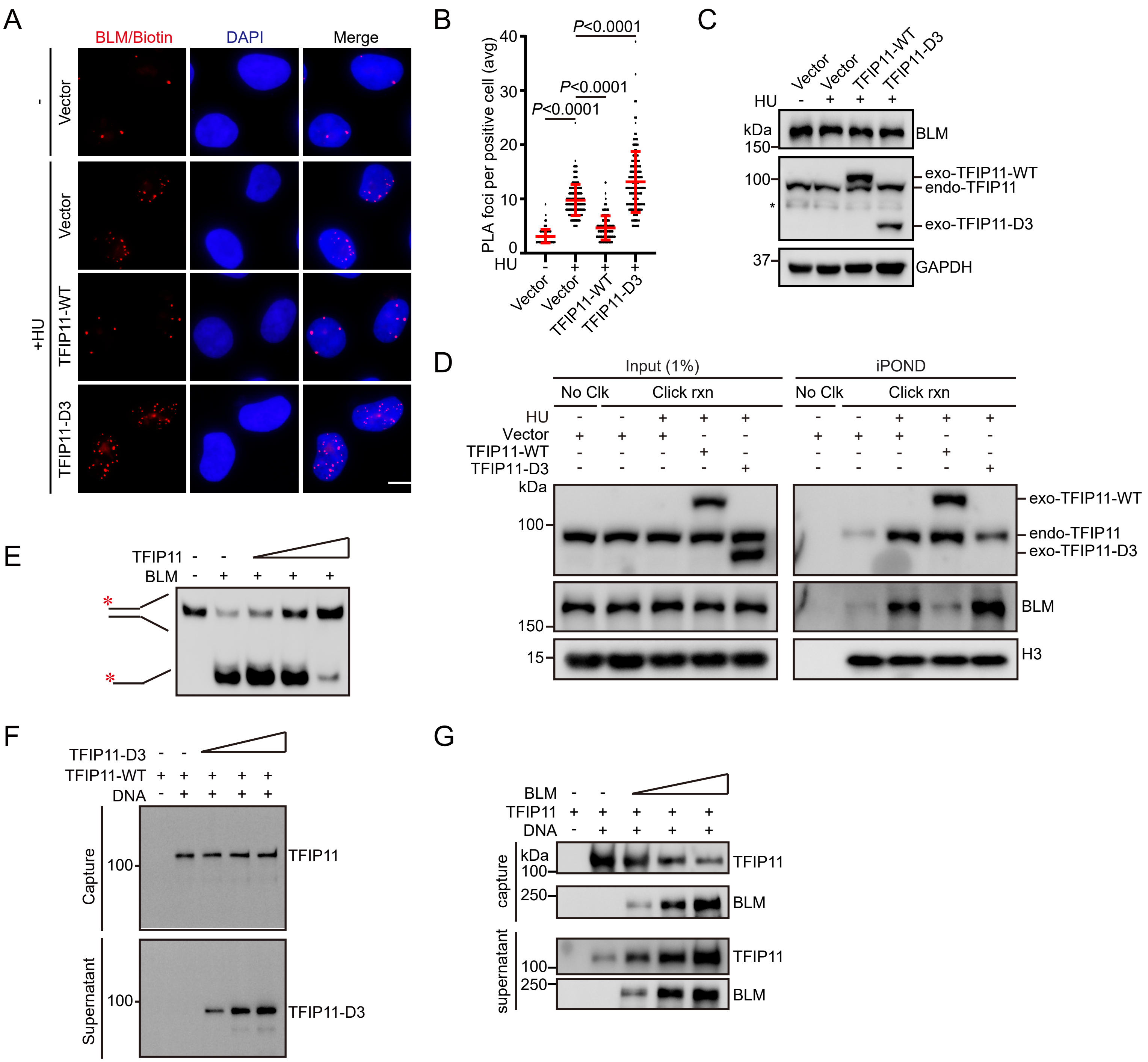
A
BLM/Biotin
DAPI
Merge
-
Vector
+HU
Vector
TFIP11-WT
TFIP11-D3
B
PLA foci per positive cell (avg)
40
30
20
10
0
P<0.0001
P<0.0001
P<0.0001
HU
- + + +
Vector
Vector
TFIP11-WT
TFIP11-D3
C
Vector
Vector
TFIP11-WT
TFIP11-D3
HU - + + +
kDa
150
BLM
100
*
exo-TFIP11-WT
endo-TFIP11
exo-TFIP11-D3
37
GAPDH
D
Input (1%)
iPOND
No Clk
Click rxn
No Clk
Click rxn
HU - - + + + - - + + +
Vector + + + - - + + + - -
TFIP11-WT - - - + - - - - + -
TFIP11-D3 - - - - + - - - - +
kDa
100
exo-TFIP11-WT
endo-TFIP11
exo-TFIP11-D3
150
BLM
15
H3
E
TFIP11 - -
BLM - + + + +
*
*
F
TFIP11-D3 - -
TFIP11-WT + + + + +
DNA - + + + +
Capture
100
TFIP11
Supernatant
100
TFIP11-D3
G
BLM - -
TFIP11 + + + + +
DNA - + + + +
capture
kDa
100
TFIP11
250
BLM
supernatant
100
TFIP11
250
BLM

Supplementary Figure 9. TFIP11 antagonizes BLM at stalled forks.

(A-C) Overexpression of TFIP11 inhibits BLM/biotin PLA focus formation. PLA was conducted with anti-BLM and anti-biotin antibodies. Representative images of PLA foci (red) were shown in (A). DNA was stained with DAPI. Scale bar, 10 μm. Quantification of the average number of PLA foci per focus positive cell (B). Data represent mean ± SD from three independent experiments. From left, $n = 147$, 147, 168, 147 cells. P values were derived from a one-way ANOVA with Tukey's multiple comparisons test. Western blot analysis of TFIP11 expression (C). Asterisk indicates a non-specific band. **(D)** Input and iPOND samples were analyzed by Western blotting. **(E)** Unwinding of 1 nM splayed fork catalyzed by BLM (20 nM) in the presence of increasing amounts of TFIP11 (10 nM, 20 nM, 40 nM). **(F)** Wild-type TFIP11 (24 nM) was added to biotinylated splayed-arm DNA (100 nM) coupled to magnetic streptavidin beads in binding buffer (20 mM Tris-HCl, PH 7.5, 120 mM NaCl, 0.1% Triton X-100, 2 mM $CaCl_2$, 10 mM $Mg(OAc)_2$, 1 mM DTT, 0.1 mg/mL BSA) at 4 °C for 30 min. Excess TFIP11 was removed by magnetic separation. Then, increasing amounts of TFIP11-D3 (12 nM, 24 nM, 48 nM) was added and reactions were incubated at 4 °C for 30 minutes prior to separation of the DNA-bound and supernatant fractions and analysis by SDS-PAGE. **(G)** TFIP11 (24 nM) was added to biotinylated splayed-arm DNA (100 nM) coupled to magnetic streptavidin beads in 20 μl of binding buffer (20 mM Tris-HCl, PH 7.5, 120 mM NaCl, 0.1% Triton X-100, 2 mM $CaCl_2$, 10 mM $Mg(OAc)_2$, 1 mM DTT, 0.1 mg/mL BSA) at 4 °C for 30 min. Excess TFIP11 was removed by magnetic separation. Then, increasing amounts of BLM (12 nM, 24 nM, 48 nM) was added and reactions were incubated at 4 °C for 30 minutes prior to separation of the DNA-bound and supernatant fractions and analysis by SDS-PAGE. Source data are provided as a Source Data file.

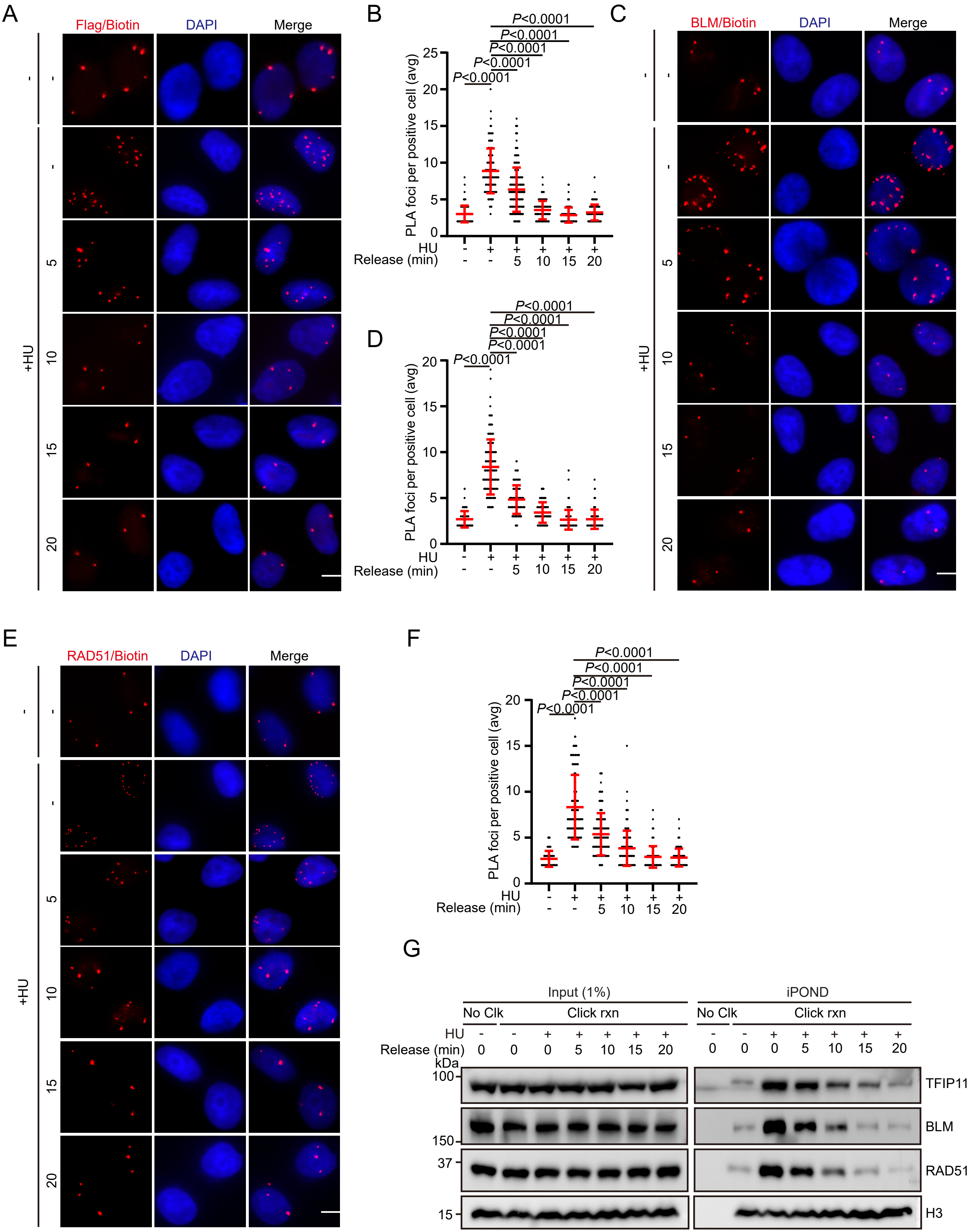
A
Flag/Biotin
DAPI
Merge
+HU
5
10
15
20
B
P<0.0001
PLA foci per positive cell (avg)
HU
Release (min)
C
BLM/Biotin
DAPI
Merge
D
E
RAD51/Biotin
F
G
Input (1%)
iPOND
No Clk
Click rxn
kDa
100
150
37
15
TFIP11
BLM
RAD51
H3

Supplementary Figure 10. TFIP11 rapidly dissociates from stalled forks following stress removal.

(A, C, E) SF-TFIP11 knock-in HeLa cells (A) or HeLa cells (C, E) were labeled with 10 μM EdU for 15 min before treatment with 4 mM HU for 3 h, followed by release for indicated times. PLA was conducted with anti-Flag, anti-RAD51, or anti-BLM in conjunction with anti-biotin antibodies. Representative images of PLA foci (red) were shown. DNA was stained with DAPI. Scale bar, 10 μm. Quantification of the average number of PLA foci per focus-positive cell. **(B, D)** Data represent mean ± SD from three independent experiments. From left, *n* = 168, 168, 168, 168, 168, 189 cells. *P* values were derived from a one-way ANOVA with Tukey's multiple comparisons test. **(F)** Data represent mean ± SD from three independent experiments. From left, *n* = 168, 189, 168, 216, 189, 189 cells. *P* values were derived from a one-way ANOVA with Tukey's multiple comparisons test. **(G)** Input and iPOND samples were analyzed by Western blotting. Source data are provided as a Source Data file.

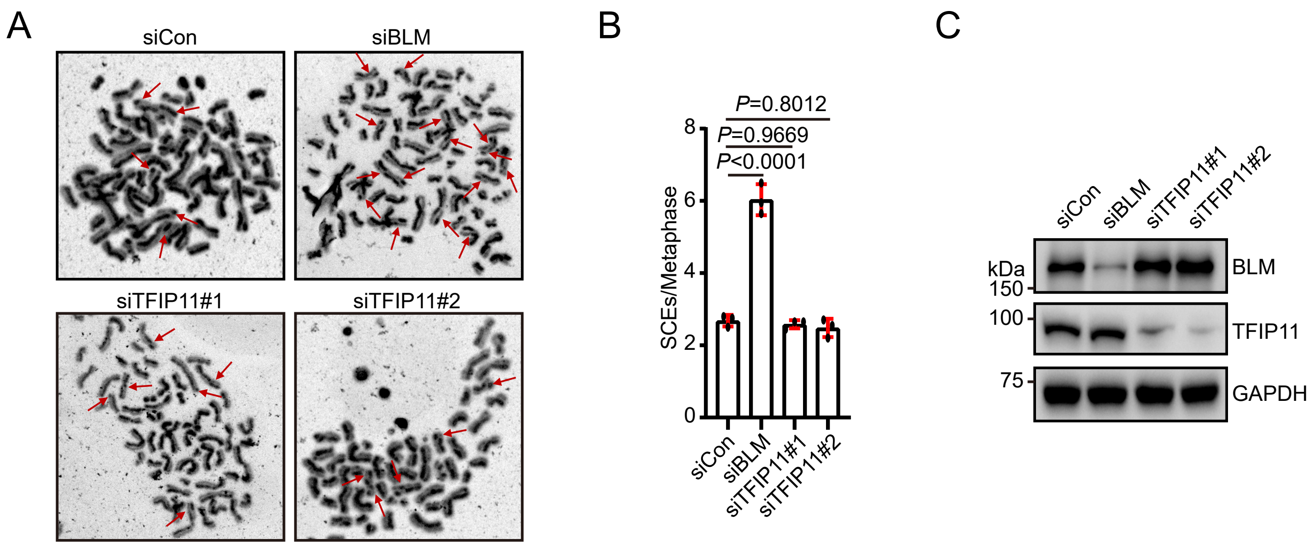

A
siCon
siBLM
siTFIP11#1
siTFIP11#2
B
P=0.8012
P=0.9669
P<0.0001
SCEs/Metaphase
8
6
4
2
0
siCon
siBLM
siTFIP11#1
siTFIP11#2
C
siCon
siBLM
siTFIP11#1
siTFIP11#2
kDa
150
100
75
BLM
TFIP11
GAPDH

Supplementary Figure 11. TFIP11 depletion has no effect on the frequency of SCE.

(A-C) Representative metaphase spreads showing SCEs from HeLa cells transfected with indicated siRNAs (red arrows) (A). Data represent mean ± SD from three independent experiments (n=3). SCEs were scored for 60 metaphase spreads for each group (B). ns, not significant; ****P<0.0001, one-way ANOVA test. Knock-down efficiency was confirmed by immunoblotting (C). Source data are provided as a Source Data file.
