# Supplementary material for: TFIP11 promotes replication fork reversal to preserve genome stability
Source: Nat Commun. 2024 Feb 10;15:1262. doi: 10.1038/s41467-024-45684-3 (PMC10858868; doi:10.1038/s41467-024-45684-3)
Supplement: Supplementary file 3 — Description of Additional Supplementary Files [file 41467_2024_45684_MOESM3_ESM.pdf]

Description of Additional Supplementary Files

File Name: Supplementary Data 1
Description: Mass spectrometry analysis of TFIP11 TAP products.
