# Supplementary material for: TFIP11 promotes replication fork reversal to preserve genome stability
Source: Nat Commun. 2024 Feb 10;15:1262. doi: 10.1038/s41467-024-45684-3 (PMC10858868; doi:10.1038/s41467-024-45684-3)
Supplement: Supplementary file 5 — Reporting Summary [file 41467_2024_45684_MOESM5_ESM.pdf]

nature portfolio

Corresponding author(s): Ting Liu

Last updated by author(s): Dec 29, 2023

Reporting Summary

Nature Portfolio wishes to improve the reproducibility of the work that we publish. This form provides structure for consistency and transparency in reporting. For further information on Nature Portfolio policies, see our Editorial Policies and the Editorial Policy Checklist.

Statistics

For all statistical analyses, confirm that the following items are present in the figure legend, table legend, main text, or Methods section.

n/a	Confirmed	
☐	☒	The exact sample size (*n*) for each experimental group/condition, given as a discrete number and unit of measurement
☐	☒	A statement on whether measurements were taken from distinct samples or whether the same sample was measured repeatedly
☐	☒	The statistical test(s) used AND whether they are one- or two-sided<br>*Only common tests should be described solely by name; describe more complex techniques in the Methods section.*
☒	☐	A description of all covariates tested
☒	☐	A description of any assumptions or corrections, such as tests of normality and adjustment for multiple comparisons
☐	☒	A full description of the statistical parameters including central tendency (e.g. means) or other basic estimates (e.g. regression coefficient) AND variation (e.g. standard deviation) or associated estimates of uncertainty (e.g. confidence intervals)
☐	☒	For null hypothesis testing, the test statistic (e.g. *F*, *t*, *r*) with confidence intervals, effect sizes, degrees of freedom and *P* value noted<br>*Give P values as exact values whenever suitable.*
☒	☐	For Bayesian analysis, information on the choice of priors and Markov chain Monte Carlo settings
☒	☐	For hierarchical and complex designs, identification of the appropriate level for tests and full reporting of outcomes
☒	☐	Estimates of effect sizes (e.g. Cohen's *d*, Pearson's *r*), indicating how they were calculated

Our web collection on statistics for biologists contains articles on many of the points above.

Software and code

Policy information about availability of computer code

Data collection	Images were captured using a Nikon Eclipse 80i Fluorescence Microscope equipped with a Plan Fluor 60 × oil objective lens (numerical aperture [NA] 0.5–1.25; Nikon) and a camera (CoolSNAP HQ2; Photometrics) and analyzed with NIS-Elements basic research imaging software (Nikon).
Data analysis	The GraphPad Software and Image J for quantitative and statistical analysis. Adobe Photoshop and Illustrator were used to process data for publication.

For manuscripts utilizing custom algorithms or software that are central to the research but not yet described in published literature, software must be made available to editors and reviewers. We strongly encourage code deposition in a community repository (e.g. GitHub). See the Nature Portfolio guidelines for submitting code & software for further information.

Data

Policy information about availability of data

All manuscripts must include a data availability statement. This statement should provide the following information, where applicable:

- Accession codes, unique identifiers, or web links for publicly available datasets
- A description of any restrictions on data availability
- For clinical datasets or third party data, please ensure that the statement adheres to our policy

The Mass spectrometry data have been deposited to the ProteomeXchange Consortium via the PRIDE partner repository under the accession code PXD042222.

Uncropped immunoblotting images are provided in a Source Data file. The raw data used in plots and graphs are all provided in a Source Data file. Source data are provided with this paper.

Research involving human participants, their data, or biological material

Policy information about studies with human participants or human data. See also policy information about sex, gender (identity/presentation), and sexual orientation and race, ethnicity and racism.

Reporting on sex and gender	N/A. No human research participants.
Reporting on race, ethnicity, or other socially relevant groupings	N/A. No human research participants.
Population characteristics	N/A. No human research participants.
Recruitment	N/A. No human research participants.
Ethics oversight	N/A. No human research participants.

Note that full information on the approval of the study protocol must also be provided in the manuscript.

Field-specific reporting

Please select the one below that is the best fit for your research. If you are not sure, read the appropriate sections before making your selection.

☒ Life sciences ☐ Behavioural & social sciences ☐ Ecological, evolutionary & environmental sciences

For a reference copy of the document with all sections, see nature.com/documents/nr-reporting-summary-flat.pdf

Life sciences study design

All studies must disclose on these points even when the disclosure is negative.

Sample size	No statistical method was used to predetermine sample size, but we routinely employed at least three biological repeats.
Data exclusions	No data was excluded.
Replication	The results reported in this study were successfully replicated in at lease three independent experiments.
Randomization	Random allocation was used for sample assignment to treatment groups.
Blinding	Data acquisition and analysis were performed in a blinded manner

Reporting for specific materials, systems and methods

We require information from authors about some types of materials, experimental systems and methods used in many studies. Here, indicate whether each material, system or method listed is relevant to your study. If you are not sure if a list item applies to your research, read the appropriate section before selecting a response.

Materials & experimental systems

n/a	Involved in the study	
☐	☒	Antibodies
☐	☒	Eukaryotic cell lines
☒	☐	Palaeontology and archaeology
☒	☐	Animals and other organisms
☒	☐	Clinical data
☒	☐	Dual use research of concern
☒	☐	Plants

Methods

n/a	Involved in the study	
☒	☐	ChIP-seq
☐	☒	Flow cytometry
☒	☐	MRI-based neuroimaging

Antibodies

Antibodies used	Polyclonal anti-TFIP11, anti-RAD51, anti-BLM and anti-RPA2 were generated by immunizing rabbits with MBP-TFIP11 full length, MBP-BLM, and MBP-RPA2 full length fusion proteins expressed and purified from E.Coli. Antisera were affinity-purified using the

	AminoLink Plus immobilization and purification kit (Thermo Fisher Scientific). Anti-Myc(M20002S) and anti-CtIP(61141) antibodies were purchased from Abmart and Active Motif, respectively. Anti-CldU/BrdU (ab6326) and anti-H3 (04-928) antibodies were purchased from Abcam and EMD Millipore, respectively. Anti-PCNA (PC10) (sc-56) and anti-Flag (M2) antibodies were purchased from Santa Cruz Biotechnology and Sigma-Aldrich, respectively. Anti-BRCA2(A303-434A), anti-Biotin (150-109A) and anti-RMI1 (A300-631A), and anti-BRCA1 (A301-378A) antibodies were purchased from Bethyl. Anti-IdU/BrdU (B44) (347580) antibody was purchased from BD Biosciences. Rhodamine conjugated goat antimouse IgG (15-001-003) and anti-biotin (200-002-211) antibodies were purchased from Jackson ImmunoResearch. Alexan Fluor 488 Donkey anti-Rat IgG (A-21208) was purchased from Life technologies. Anti-BrdU (RPN202) was purchased from GE.
Validation	All commercial anti-bodies are validated by manufacturers via western blot analysis and immunofluorescence as documented in the manufacturer's websites.

Eukaryotic cell lines

Policy information about cell lines and Sex and Gender in Research

Cell line source(s)	HeLa, HEK293T and U2OS cells were from ATCC.
Authentication	N/A
Mycoplasma contamination	All cell lines used in this study have been cultured in the presence of anti-mycoplasma antibiotics (BioMycoX Mycoplasma Elimination kit, BioInd) to prevent mycoplasma contamination. All cells were confirmed mycoplasma negative.
Commonly misidentified lines (See ICLAC register)	No commonly misidentified cell lines were employed in this study.

Flow Cytometry

Plots

Confirm that:

☒ The axis labels state the marker and fluorochrome used (e.g. CD4-FITC).

☒ The axis scales are clearly visible. Include numbers along axes only for bottom left plot of group (a 'group' is an analysis of identical markers).

☒ All plots are contour plots with outliers or pseudocolor plots.

☒ A numerical value for number of cells or percentage (with statistics) is provided.

Methodology

Sample preparation	For BrdU incorporation assays, U2OS cells were transfected with the indicated siRNAs. 48 hr after transfection, BrdU were added into the medium for 1 hr. Cells were then harvested and fixed with ice-cold 70% ethanol. DNA was denatured with 2.5 M HCl for 1 hr at room temperature. After washing with PBS, cells were incubated in mouse anti-BrdU antibody in blocking buffer (PBS + 0.1% Triton X-100 + 5% BSA) for 12 hr followed by washing with blocking buffer containing 500 mM NaCl. FITC conjugated goat anti-mouse IgG was added and incubated for 4 hr. Cells were then resuspended in PBS containing propidium iodide (20 µg/mL) and RNase A (200 µg/mL) at 37°C for 20 min. For HR assays, U2OS DR-GFP cells were transfected with indicated siRNAs and 48 h later were electroporated with an I-SceI expression plasmid. 48 h after electroporation, cells were harvested and assayed for GFP expression by flow cytometry analysis.
Instrument	FACScan flow cytometer (Beckman)
Software	FlowJo software was used to analyze the data.
Cell population abundance	Not applicable to this study.
Gating strategy	Cells were gated for GFP population as an output for HR efficiency.

☒ Tick this box to confirm that a figure exemplifying the gating strategy is provided in the Supplementary Information.
